# Homeopathy for Covid-19 in Primary Care: A structured summary of a study protocol for a randomized controlled trial

**DOI:** 10.1186/s13063-021-05071-5

**Published:** 2021-02-01

**Authors:** Ubiratan Cardinalli Adler, Maristela Schiabel Adler, Livia Mitchiguian Hotta, Ana Elisa Madureira Padula, Amarilys de Toledo Cesar, José Nelson Martins Diniz, Crislaine Aparecida Antonio Mestre, Katia Regina Spiller, Lidiamara Soares, Helen de Freitas Santos, Edson Zangiacomi Martinez

**Affiliations:** 1 Medicine Department, Universidade Federal de São Carlos, Rodovia Washington Luiz, Km 235, São Carlos, SP Brasil 13565-905; 2 Centro Municipal de Práticas Integrativas e Complementares em Saúde CEMPICS, Rua Joaquim Miranda, 471, Guarulhos, SP 07023-051 Brasil; 3 Universidade Presbiteriana Mackenzie, R. Piauí, 181, São Paulo, SP 01241-001 Brasil; 4 Instituto HN-Cristiano, Rua Dr. Cesar 212, São Paulo, SP 02013-001 Brasil; 5 Universidade Federal de São Carlos, School Health Unit (USE), Rodovia Washington Luiz, Km 235, São Carlos, SP Brasil 13565-905; 6 São Carlos’ Health Surveillance, R. Conde do Pinhal, 2161 –, São Carlos, SP 13560-648 Brasil; 7 São Carlos’ Epidemiological Surveillance, R. Conde do Pinhal, 2161, São Carlos, SP 13560-648 Brasil; 8 Instituto Federal de Educação Ciência e Tecnologia de São Paulo, R. Pedro Cavalo, 709, Birigui, SP 16201-407 Brasil; 9 Social Medicine Department, Universidade de São Paulo - Faculdade de Medicina de Ribeirão Preto, Av. Bandeirantes, 3900, Ribeirão Preto, SP Brasil 14049-900

**Keywords:** COVID-19, Homeopathy, Unified Health System, Primary Care, Telemedicine, Randomized Controlled Trial protocol

## Abstract

**Objectives:**

To investigate the effectiveness and safety of homeopathic medicine *Natrum muriaticum* (LM2) for mild cases of COVID-19 in Primary Health Care.

**Trial design:**

A randomized, two-armed (1:1), parallel, placebo-controlled, double-blind, clinical trial is being performed to test the following hypotheses:

- H0: homeopathic medicines = placebo (null hypothesis) vs.
- H1: homeopathic medicines ≠ placebo (alternative hypothesis) for mild cases of COVID-19 in Primary Care.

**Participants:**

Setting: Primary Care of São Carlos – São Paulo – Brazil.

One hundred participants aged 18 years or older, with Influenza-like symptoms and a positive RT-PCR for SARS-CoV-2. Willingness to give informed consent and to comply with the study procedures is also required. Exclusion criterium: severe acute respiratory syndrome.

**Intervention and comparator:**

- Homeopathy: 1 globule of Natrum muriaticum LM2 diluted in 20 mL of alcohol 30% and dispensed in a 30 ml bottle.
- Placebo: 20 mL of alcohol 30% dispensed in a 30 ml bottle.

Posology: one drop taken orally every 4 hours (6 doses/day) while there is fever, cough, tiredness, or pain (headache, sore throat, muscle aches, chest pain, etc.) followed by one drop every 6 hours (4 doses/day) until the fourteenth day of use. The bottle of study medication should be submitted to 10 vigorous shakes (succussions) before each dose. Posology may be changed by telemedicine, with no break in blinding.

Study medication should be maintained during home isolation. According to the Primary Care protocol, the home isolation period lasts until the 10^th^ day after the appearance of the first symptom, or up to 72 hours without symptoms.

**Main outcomes:**

The primary endpoint will be time to recovery, defined as the number of days elapsed before all COVID-19 Influenza-like symptoms are recorded as mild or absent during home isolation period. Secondary measures are recovery time for each COVID-19 symptom; score of the scale created for the study (COVID-Simile Scale); medicines used during follow-up; number of days of follow-up; number of visits to emergency services; number of hospitalizations; other symptoms and Adverse Events during home isolation period.

**Randomisation:**

The study Statistician generated a block randomization list, using a 1:1 ratio of the two groups (denoted as A and B) and a web-based tool (http://www.random.org/lists).

**Blinding (masking):**

The clinical investigators, the statistician, the Primary Care teams, the study collaborators, and the participants will remain blinded from the identity of the two treatment groups until the end of the study.

**Numbers to be randomised (sample size):**

One hundred participants are planned to be randomized (1:1) to placebo (50) or homeopathy (50).

**Trial Status:**

Protocol version/date May 21, 2020. Recruitment is ongoing. First participant was recruited/included on June 29,2020. Due to recruitment adaptations to Primary Care changes, the authors anticipate the trial will finish recruiting on April 10, 2021.

**Trial registration:**

COVID-Simile Study was registered at the University Hospital Medical Information Network (UMIN - https://www.umin.ac.jp/ctr/index.htm) on June 1^st^, 2020, and the trial start date was June 15, 2020. Unique ID: UMIN000040602.

**Full protocol:**

The full protocol is attached as an additional file, accessible from the Trials website (Additional file 1). In the interest in expediting dissemination of this material, the familiar formatting has been eliminated; this Letter serves as a summary of the key elements of the full protocol.

**Supplementary Information:**

The online version contains supplementary material available at 10.1186/s13063-021-05071-5.

## Supplementary Information

**Additional file 1.**

## Data Availability

The data will be available from the author on reasonable request. Ubiratan Cardinalli Adler, M. D, Ph.D. Universidade Federal de São Carlos, Medicine Department Rodovia Washington Luiz, Km 235, São Carlos, SP, Brasil – 13565-905 Tel. +55 16 3351-9420 ubiratanadler@ufscar.br

