# Supplementary material for: Homeopathy for Covid-19 in Primary Care: A structured summary of a study protocol for a randomized controlled trial
Source: Trials. 2021 Feb 1;22:109. doi: 10.1186/s13063-021-05071-5 (PMC7848248; doi:10.1186/s13063-021-05071-5)
Supplement: Supplementary file 1 — Additional file 1. [file 13063_2021_5071_MOESM1_ESM.pdf]

Homeopathy for Covid-19 in Primary Care: a study protocol for a randomized controlled trial (Covid-Simile study protocol)

December 2020

ASSOCIATED RESEARCHERS

Ubiratan Cardinalli Adler, Ph.D.

Universidade Federal de São Carlos, Medicine Department

Rodovia Washington Luiz, Km 235, São Carlos, SP, Brasil – 13565-905

Tel. +55 16 3351-9420

ubiratanadler@ufscar.br - **Corresponding author**

Maristela Schiabel Adler, Ph.D.

Universidade Federal de São Carlos, Medicine Department

Rodovia Washington Luiz, Km 235, São Carlos, SP, Brasil – 13565-905

msadler@ufscar.br

Livia Mitchiguian Hotta, MD

Centro Municipal de Práticas Integrativas e Complementares em Saúde

CEMPICS

Rua Joaquim Miranda, 471, Guarulhos - SP, 07023-051

livia.m.hotta@terra.com.br

Ana Elisa Madureira Padula, M.Sc.

Universidade Presbiteriana Mackenzie

R. Piauí, 181 – São Paulo – SP, 01241-001

anapadula@uol.com.br

Amarilys de Toledo Cesar, Ph.D.

Instituto HN-Cristiano

Rua Dr. Cesar 212, São Paulo – SP, 02013-001

amarilys@hncristiano.com.br

José Nelson Martins Diniz, Ph.D.

Universidade Federal de São Carlos, School Health Unit (USE)

Rodovia Washington Luiz, Km 235, São Carlos, SP, Brasil – 13565-905

jnmdiniz@gmail.com

Crislaine Aparecida Antonio Mestre, R.N.

São Carlos' Health Surveillance

R. Conde do Pinhal, 2161 – São Carlos, SP, 13560-648

crislaine.mestre@saocarlos.sp.gov.br

Katia Regina Spiller, R.N.

São Carlos' Epidemiological Surveillance

R. Conde do Pinhal, 2161 – São Carlos, SP, 13560-648

kakaspiller@gmail.com

Lidiamara Soares, R.N.

São Carlos' Epidemiological Surveillance

R. Conde do Pinhal, 2161 – São Carlos, SP, 13560-648

lindiamaras@yahoo.com.br

Helen de Freitas Santos, Ph.D.

Instituto Federal de Educação Ciência e Tecnologia de São Paulo

R. Pedro Cavalo, 709, Birigui – SP, 16201-407

helen.ifsp.bri@gmail.com

Edson Zangiacomi Martinez, Ph.D

Universidade de São Paulo - Faculdade de Medicina de Ribeirão Preto

Social Medicine Department

Av. Bandeirantes, 3900, Ribeirao Preto, SP, Brasil – 14049-900

edson@fmrp.usp.br

KEY WORDS

COVID-19; Homeopathy; Unified Health System; Primary Care; Telemedicine; Randomized Controlled Trial protocol

ABSTRACT

Objective: To investigate the effectiveness and safety of homeopathic medicine *Natrum muriaticum* (LM2) for mild cases of COVID-19 in Primary Health Care. **Design:** randomized, parallel-group, double-blind, placebo-controlled, clinical trial. **Setting:** Primary Care of São Carlos – São Paulo – Brazil. **Subjects:** 100 participants aged 18 years or older, with Influenza-like symptoms and a positive RT-PCR for SARS-CoV-2. Willingness to give informed consent and to comply with the study procedures will also be required. Exclusion criterium: severe acute respiratory syndrome. **Interventions:** one drop of a 30% hydroalcoholic solution of *Natrum muriaticum* LM2 or of an indistinguishable placebo (30% hydroalcoholic) solution taken orally every 4 hours (6 doses/day) while there is fever, cough, tiredness, or pain (headache, sore throat, muscle aches, chest pain, etc.) followed by one drop every 6 hours (4 doses/day) during home isolation (10th day after the onset of symptoms, or up to 72 hours without symptoms). The bottle of study medication should be submitted to 10 vigorous shakes (succussions) before each dose. **Outcome Measures:** The primary endpoint will be time to recovery, defined as the number of days elapsed before all COVID-19 Influenza-like symptoms are recorded as mild or absent during home isolation period. Secondary measures: recovery time for each COVID-19 symptom; score of the scale created for the study (COVID-Simile Scale); number of days of follow-up; medicines used during follow-up; number of visits to emergency services; number of hospitalizations; other symptoms and Adverse Events during home isolation period. **Discussion**: COVID-Simile is the only double-blinded, randomized, placebo-controlled clinical trial planned to evaluate a homeopathic strategy for COVID-19 in Primary Care. If results refute the null hypothesis, exactly prepared homeopathic *Natrum muriaticum* LM2 could be incorporated into the clinical management of COVID-19 in Primary Care, in parallel with larger studies to confirm its effectiveness.

INTRODUCTION

Nine months after the first COVID-19 reported case on February 26th, Brazil is rapidly approaching 7.5 million cases and 190 thousand deaths [1]. The Brazilian state of São Paulo, the epicenter of the disease in the country, has the highest death toll [2].

Located in the geographic center of São Paulo, São Carlos is a mid-sized university town (ca. 250 thousand inhabitants[3]). The coronavirus pandemic reached the town on March 18, 2020[4], when we began to study homeopathic strategies for coping with the disease. Figure 1 shows biweekly reported COVID-19 total cases (A), deaths (B) and new cases (C) in São Carlos/São Paulo[5] since the case index, stressing an acceleration in new cases since November 2020.

Figure 1 – Biweekly reported COVID-19 total cases, total deaths, and new cases in São Carlos/São Paulo/Brazil

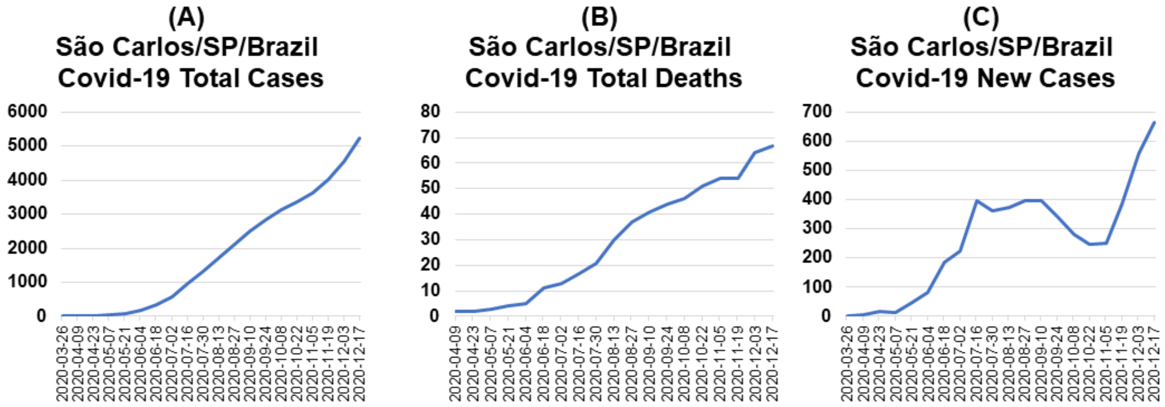

Source: São Carlos' Emergency Coronavirus Combat Committee

Non-conventional research in the Brazilian Unified Health System (SUS) is encouraged by the National Policy on Integrative and Complementary Health Practice[6]. Homeopathy (a medical specialty in Brazil[7]) was developed by Samuel Hahnemann during 45 years of pharmacoclinical experiments. He improved the pharmacology and posology of homeopathy, until reaching what he considered to be his "most perfected method"[8]: repeated (as needed) doses of the "best chosen" homeopathic medicine in liquid form (so that it could be succussed before each dose[9]), prepared trough the fifty-millesimal dynamization[10] and used in ascending potencies (when needed), beginning with the lowest degrees[6].

Fifty-millesimal or Quinquagintamillesimal (LM or Q) potencies are prepared by trituration of the raw material (in three 1:100 steps), followed by consecutive 1:50,000 succussed dilutions. Therefore, a LM1 potency corresponds to $5x10^{-10}$ fraction of the raw material (LM2 = 2.5×10^{-15}, LM3 = 1.25×10^{-20}, LM4 = 6.25×10^{-24} etc.). Hahnemann's 18th century dynamizing or potentizing, i.e., the mechanical action upon particles of the raw material, separated from each other through the addition of an indifferent medium, dry (for trituration) of fluid (for dilution)[11], have been recognized as a manual method to produce nanoparticles of the source material[12].

According to Hahnemann's instructions, the finding of a homeopathically suitable remedy for an epidemic disease requires the examination of several patients to identify the peculiarities of the collective disease, i.e., the *"more marked and special symptoms which are peculiar to but few diseases and of rarer occurrence, at least in the same combination"* [13]. General symptoms (loss of appetite, headache, debility, sleeplessness, discomfort, etc.), on the other

hand, demand but little attention, if they cannot be more accurately described and present no peculiarities, *"as symptoms of such a general nature are observed in almost every disease and from almost every drug"* [14] .

When COVID-19 reached São Carlos, we had no patients to examine. A literature search at that time led us to the interesting findings of the German virologist Hendrik Streeck: ca. of 2/3 of more than 100 patients affected by COVID-19 had described a long-lasting (many days) loss of smell and taste[15]. Later, anosmia and ageusia proved to be COVID-19 symptoms reported in different countries [16-17].

Coincidentally, Hahnemann himself came across an epidemic fever in 1809 and ageusia was one of the characteristic symptoms stressed by him: *"on taking any food, the sense of taste appears as if extinguished"*. Ageusia is also a pathogenetic symptom of the two medicines Hahnemann considered most appropriate to that epidemic fever: *Nux vomica* and *Arsenicum album*, this one chosen because it "was capable of exciting those symptoms which make up the fever in a more perfect manner" [18].

In collaboration with colleagues from the city of São Paulo, we had the opportunity to contact six suspected or confirmed cases of COVID-19. All of them reported anosmia and/or ageusia among other symptoms that we considered characteristic: "breathing was short, almost breathless"; dry and constant cough; cough when speaking (making it difficult to report symptoms over the phone); "cough when you get out of bed and move around".

After identifying the peculiarities of the collective disease COVID-19, we move on to the next step, the selection of the most suitable homeopathic medicine. According to Hahnemann, *"the search for and selection of the homeopathic remedy most suitable in every respect to each morbid state, is an operation which, notwithstanding all the admirable books for facilitating it, still demands the study of the original sources themselves"* [19]. The "original sources", mentioned by him, are collections of symptoms (or "Materia Medica") obtained mainly from drug experiments on healthy volunteers, symptoms nowadays denominated as "pathogenetic"[20].

We searched the original sources using the Materia Medica search function of Vision software[21]. Our findings indicated *Thuja occidentalis* and *Natrum muriaticum* as the most homeopathically suitable remedies to the symptoms we had identified. The first hypothesis, *Thuja*, was given in repeated doses (*Thuja* LM2) to three adult patients from São Paulo city, by telemedicine. The patients did recover, but the disease seemed to have run its course, with no sign of rapid improvement, as we would be expected in acute cases[22]. We then proceeded to the second hypothesis, *Natrum muriaticum* (Nat-m).

Pathogenetic symptoms that led us to consider Nat-m as homeopathic to COVID-19 were found (via Vision software) in Hahnemann's "The Chronic Diseases"[23] and Allen's "The Encyclopedia of Pure Materia Medica" [24] :

- "Moderate coryza, with total loss of smell and taste" (Hahnemann/Chronic Diseases/Nat-m # 811) and "Excessive fluent coryza, with loss of all power of smell and taste" (Hahnemann/Chronic Diseases/Nat-m # 812);
- "Incitement to coughing and tightness of the chest from speaking"

(Hahnemann/Chronic Diseases/Nat-m # 827);

- "Cough when walking" (Hahnemann/Chronic Diseases//Nat-m/Introduction);
- "Shortness of breath" (Allen/Encyclopedia/ Nat-m # 1810) and "Oppression of the chest, and shortness of breath". (Allen/Encyclopedia/ Nat-m # 1855);

From March 29th to May 10th, we treated 10 consecutive cases of COVID-19 with Nat-m LM2 by telemedicine, which had been recently regulated by the Brazilian Ministry of Health to deal with the COVID-19 pandemic[25]. Of the 10 cases, 6 are women and 4 men, with a mean age of 37.8 years (24–56 years). They had manifested COVID-19 symptoms 8 days (2-14 days) before the first dose. All presented a recent reduction and/or loss of taste and/or smell; 2 had a positive RT-PCR for SARS-CoV-2; another 3 patients had characteristic COVID-19 chest computed tomography images. Patients could use other medications along with Nat-m: one used azithromycin and oseltamivir; another oseltamivir alone and three patients took analgesics. They kindly agreed to fill in a daily follow-up form until symptom remission. We have named that form as "COVID-Simile Scale" (Appendix 1), aware that it was not a validated scale, but a simple clinical diary, that helped us standardizing the assessment of home isolated patients. Based on patients' reports we could infer a mean daily score (Figure 2).

Figure 2 - Daily mean clinical score (based on "COVID-Simile scale") of 10 COVID-19 cases treated with Nat-m LM2

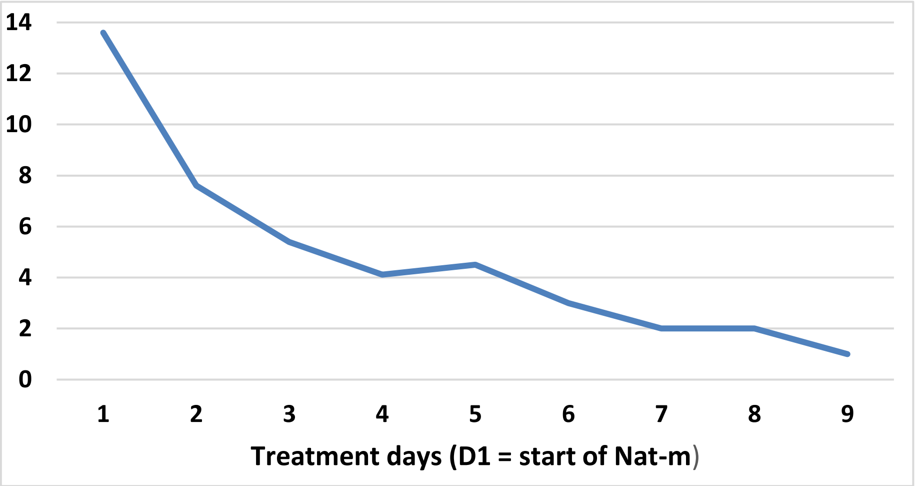

Although anosmia and ageusia are complaints that took relatively longer to improve, that small sample of patients showed a rapid improvement, reinforcing our hypothesis that *Natrum muriaticum* might contribute to a faster recovery of COVID-19. To test it, we have decided to run a clinical study.

We chose Nat-m prepared in a fifty-millesimal potency, not only because it is the recommended dynamization by Hahnemann, but also by our experience using LM potencies in randomized controlled clinical trials [26] [27] [28] [29]. Our option for Primary Care stemmed from the possibility of treating milder cases.

SUS Primary Care unities of São Carlos are responsible for screening, treating, and telemonitoring patients with Influenza-like illness during home isolation and referring severer cases to hospitalization. PCR swabs were initially collected in all Primary Care units, later on in regional Primary Care units, but due to logistic

problems, SARS-Cov-2 PCR-collection in São Carlos is currently restricted to five Primary Care units and at a reference center. Sample processing is carried out at the Federal University of São Carlos (UFSCar) laboratories, which post the results on the website of São Carlos' Epidemiological Surveillance.

Primary Care teams have been informing PCR results to their patients, but since November 2020 patients can access their PCR-result page. COVID-19 screening, testing, treatment, and monitoring are government (SUS) funded. COVID-Simile study will add on procedures to Primary Care COVID-19 routine.

OBJECTIVE

To investigate the effectiveness and safety of homeopathic medicine *Natrum muriaticum* (LM2) for mild cases of COVID-19 in Primary Care.

METHODS

Guidelines

The COVID-Simile study is conducted by the following guidelines: Regulatory Standards for Research involving Human Beings in Brazil[30]; International Council on Harmonisation of Technical Requirements for Registration of Pharmaceuticals for Human Use (ICH) [31]; National Research Ethics Commission (CONEP) for conducting research during COVID-19[32] and CONSORT (Consolidated Standards of Reporting Trials)[33].

Trial design

A randomized, two-armed (1:1), parallel, placebo-controlled, double-blind, clinical trial is being performed to test the following hypotheses:

- H0: homeopathic medicines = placebo (null hypothesis) vs.

- H1: homeopathic medicines ≠ placebo (alternative hypothesis) for mild cases of COVID-19 in Primary Care.

Study setting

SUS Primary Health Care - São Carlos – São Paulo, Brazil.

Participants

Women and men aged 18 years or older, with Influenza-like symptoms and a positive RT-PCR for SARS-CoV-2, will be included. Willingness to give informed consent and to comply with the study procedures is also required. Exclusion criterium: severe acute respiratory syndrome.

Interventions

- Homeopathy: 1 globule of *Natrum muriaticum* LM2 diluted in 20 mL of alcohol 30% and dispensed in a 30 ml bottle.
- Placebo: 20 mL of alcohol 30% dispensed in a 30 ml bottle.

Posology: one drop taken orally every 4 hours (6 doses/day) while there is fever, cough, tiredness, or pain (headache, sore throat, muscle aches, chest pain, etc.) followed by one drop every 6 hours (4 doses/day) until the fourteenth day of use. The bottle of study medication should be submitted to 10 vigorous shakes (succussions) before each dose. Posology may be changed by telemedicine, with no break in blinding.

Study medication should be maintained during home isolation. According to the SUS Primary Care protocol, quarantine lasts until the 10th day after the appearance of the first symptom, or up to 72 hours without symptoms.

Natrum muriaticum LM2 and placebo have been kindly provided by HN-Cristiano Homeopatia (www.homeopatiahncristiano.com.br), which manufactures LM-potencies in conformity with Hahnemann standards (standardized LM potencies)[34].

Outcomes

The primary endpoint will be time to recovery, defined as the number of days elapsed before all COVID-19 Influenza-like symptoms[35] are recorded as mild or absent[36] during home isolation period. Secondary measures are: recovery time for each COVID-19 symptom; score of the scale created for the study (COVID-Simile Scale); medicines used during follow-up; number of days of follow-up; number of visits to emergency services; number of hospitalizations; other symptoms and Adverse Events. In the SUS Primary Care protocol there are no follow up PCR-tests for SARS-CoV-2.

Sample size

Considering two independent parallel groups, allocation 1: 1; effect size 0.6; Type 1 error of 5% and Type 2 error of 20% the sample size calculated by G * Power 3.1.9.2 software would be 90 participants. Predicting a 10% follow-up loss, we plan to include 100 participants with COVID-19, randomized to the two treatment arms (Figure 3).

Figure 3 - Flow diagram of participation

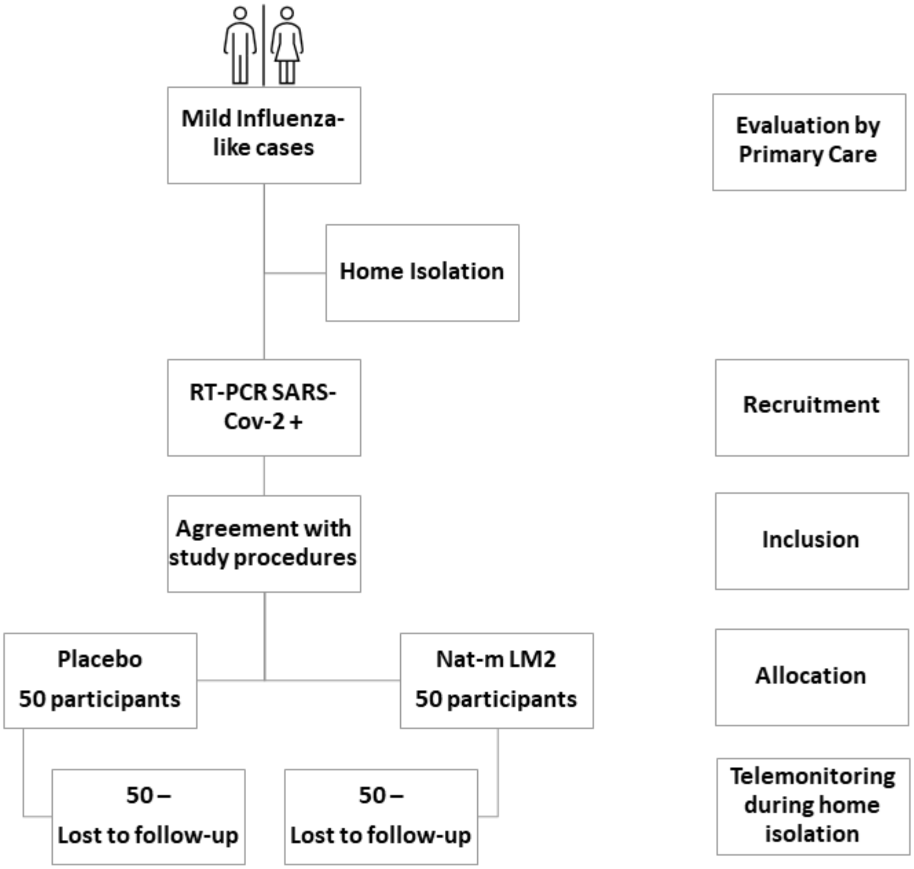

Recruitment

Primary Care follows the Clinical Management Protocol for Influenza-like Illness (ILI) and Severe Acute Respiratory Syndrome (SARS) elaborated from the Health Department of São Carlos. Moderate and severe cases are referred for hospitalization, while mild cases of ILI are monitored in home isolation [37].

Recruitment plan has changed, adapting to the changes in Primary Care. Mild suspected cases of ILI were initially recruited by Primary Care teams at the time of the RT-PCR swabs collection (within the Primary Care unity), waiting for

SARS-CoV-2 confirmation to inclusion. In a second moment, recruitment was by carried out by Primary Care teams after a positive PCR result, registered on the Epidemiological Surveillance website.

Since November/2020, when accessing the test result on the Epidemiological Surveillance website, patients with an RT-PCR positive result receive brief information that a study on homeopathy for Covid-19 is being conducted by professors of UFSCar Medical School. They have the option to get further information (and agree to receive a call from a study researcher or not), by clicking on the respective button on the webpage. Upon an affirmative answer, a study researcher (AEMP – from São Paulo/SP) makes phone/WhatsApp contact to present the study and explain the terms of the Informed Consent (IC). She sends an electronic copy of the IC to the patient and, in case of agreement with the study procedures, records participant data on the study website.

Delivery of Informed Consent (IC) and study medication and Inclusion

An Administrative Assistant at School Health Unit/UFSCar (USE) has password-controlled access to the webpage "Recruitment" of the study website. She acknowledges each newly recruited participant, who agreed with study procedures and the respective sequential Inclusion Number, sending two counterparts of the Informed Consent (pre-signed by the Principal Investigator) and the correspondent medication package (same Inclusion Number) to the participant's home address, through a delivery company, which returns the IC signed by the participant to USE, completing the Inclusion process.

Centralized randomization, allocation concealment, and blinding

In early June, the study Statistician generated a block randomization list, using a 1:1 ratio of the two groups (denoted as A and B) and a web-based tool (http://www.random.org/lists). He sent the list to the Executive Coordinator of the

UFSCar Health School Unit (UFSCar/USE), who wrote down each randomization result (A or B) on an identification card, placed each card in sequentially numbered, sealed opaque envelopes, and handed them over to the study Pharmacist. The Administrative Director of USE (Prof. Dr. Nelci Adriana Cicuto Ferreira Rocha) decided the study code, i.e., whether A or B corresponded to homeopathy or placebo, and reported that decision to the study Pharmacist.

In mid-June 2020, the study Pharmacist opened the envelope number “X” and assigned the participant with the Inclusion Number “X” to designated study group A or B (homeopathy or placebo), according to the identification card (A or B) placed in the envelope and to the study code provided by the Administrative Director of USE. The study Pharmacist dispensed each medication bottle, accordingly, packing and sealing it. He then handed the sealed packages, labeled with the participant's respective Inclusion Number, to the Administrative Assistant (Ms. Cláudia Costa) responsible for USE medical records.

The study Pharmacist is the only study collaborator who knows the study group (A or B) registered on the card of each envelope, as well as the code, defining whether A or B of the randomization list corresponds to homeopathy or placebo. The clinical investigators, the statistician, the Primary Care teams, the study collaborators, and the participants will remain blinded from the identity of the two treatment groups until the end of the study. The study Pharmacist has no knowledge or control over which participant each study medication will be sent to.

Monitoring and Telemedicine

According to the COVID-19 Clinical Management Protocol, Primary Care teams should monitor (by phone/WhatsApp) each quarantined COVID-19 patient every

48 hours, until the 10th day after the onset of symptoms, or up to 72 hours without symptoms. Primary Care teams request a presential consultation if a physical examination is needed and refer the patient to hospital care, in case of worsening symptoms.

Initially (from June 29 onwards) participants follow-up data were updated on the study website by Primary Care teams, who, however, became progressively overloaded with the resumption of routine care, in addition to the care of Covid-19 cases. To avoid data loss, we have adapted study procedures to Primary Care reality. In addition to the monitoring carried out by Primary Care, since December 9 a study researcher (AEMP – from São Paulo/SP) makes phone/WhatsApp calls to participants during quarantine (10th day after the appearance of the first symptom, or up to 72 hours without symptoms), updating follow-up data on the study website. Covid-Simile study meets the CONEP recommendations[31], not interfering with SUS routine health care. The study adds the study medication and telemedicine-monitoring of each participant to Primary Care routine.

The study Statistician will also have password-controlled access to study website Follow-up pages. If a significant superiority of any of the study's interventions is identified, he will report it to the principal investigator (with no break in blinding). This occurrence will be communicated to CONEP, which will assess the need to adapt or suspend the study.

The clinical investigators have password-controlled access to all website pages. From follow-up data, they may contact participants to discuss changes in the posology of the study medication (with no break in blinding). The participant will have telephone/WhatsApp access to the principal investigator on study medication issues. If signs of worsening are identified, the principal investigator

will contact the respective Primary Care team, suggesting presential medical reassessment, or refer the participant to emergency service, if the worsening happens outside of office hours. Study procedures are illustrated in Figure 4.

Figure 4: Study procedures

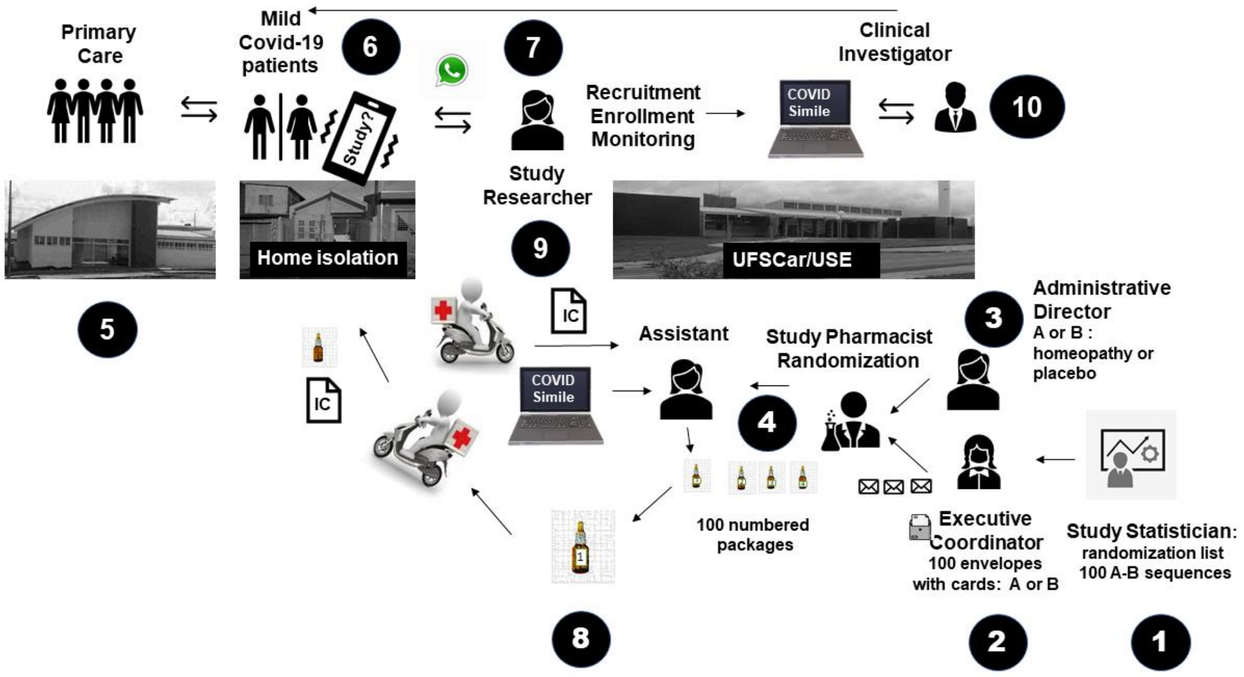

1) The study statistician generates a block randomization list of 100 A or B sequences. **2)** Executive Coordinator of the UFSCar Health School Unit (UFSCar/USE) prepared 100 A or B cards and places them in 100 sequentially numbered, sealed opaque envelopes, handing them to the study Pharmacist. **3)** The Administrative Director of UFSCar/USE decides the study code, i.e., whether A or B will correspond to homeopathy or placebo, and reports that decision on the study Pharmacist. **4)** The study Pharmacist opens the envelopes and randomizes each medicine bottle according to the letter written on each card, as well as the code communicated by the Administrative Director. He dispenses the study medication in 100 numbered and sealed packages and hands them to the Administrative Assistant. **5)** Primary Care teams treat and monitor mild Influenza-like cases in home isolation. **6)** When accessing the test result on the Epidemiological Surveillance website, patients with an RT-PCR positive result receive brief information that a study on homeopathy for Covid-19 is being conducted by professors of UFSCar Medical School. They have the option to get further information by clicking on a button on the webpage if they agree to receive a call from a study researcher. **7)** Upon an affirmative answer, the study researcher carries on patients' recruitment, participants enrollment, and monitoring, recording data at the Covid-Simile study website. **8)** The Administrative Assistant of UFSCar/USE has password-controlled access to the webpage "Enrollment" of the study website

(containing the name, inclusion number, and address of each participant) and sends the Informed Consent (IC) and respective medication package (same Inclusion Number) to the participant's home address, through a delivery company, **9)** Which brings back the signed IC to the Administrative Assistant. **10)** Clinical investigators monitor participants' progress and can adapt the posology of the study medication by telemedicine, with no break in blinding. Participants are free to contact the principal investigator on study medication questions.

Data management

Data management will be performed following the ICH-Guidelines for Good Clinical Practice. Participants' data will be inserted or accessed by password-controlled access. Follow-up clinical data will be identified by the participant' Inclusion Number, ensuring confidentiality in data monitoring and analysis. Only the Primary Care health teams, recruitment/telemonitoring researcher, and the principal investigator will have full access to the study website, i.e., to participants' identification and data.

Data analysis

Considering the time to recovery as the main outcome of the study, survival curves will be constructed using the Kaplan-Meier method, for the description of this variable. COVID-Simile Scale scores will be compared between groups by linear mixed-effects models, in which random effects are used to correlate the measurements of the same individual at different times. These models allow the insertion of confounding variables in the comparisons. Comparisons between treatment groups will use the log-rank test. In these analyzes, follow-up losses will be treated as censored information.

Hazard Ratio (HR) will be used as a measure of treatment effect, with its respective 95% confidence interval, estimated by Cox's proportional hazards model. In case the risk proportionality assumption is not met, parametric models

will alternatively be used, according to the behavior of the corresponding survival curves. Cox's model and parametric models allow the addition of other control variables for the comparison between treatment groups.

Other secondary measures, expressed as discrete variables, such as the number of hospitalizations, the number of medications will be compared between groups by generalized regression models, based on discrete measures (such as the Poisson distribution).

Outcomes expressed in qualitative variables, such as the occurrence of symptoms and adverse events, will be compared using models based on binary variables (logistic regression).

In all statistical methods, a significance level of 0.05 will be set. The analyzes will use the statistical program R version 3.6.3. A description of all mentioned statistical tools can be found in textbooks, such as Kleinbaum and Klein [38].

Administrative Authorization

COVID-Simile-Study has received written authorizations from the Secretary of Health of São Carlos, cosigned by the director of the Outpatient Management Department and by the director of Health Surveillance Department.

Independent safety-monitoring Committee

An independent data-monitoring committee has been established by the São Carlos São Carlos' Epidemiological Surveillance to assess at intervals the progress of a clinical trial, the safety data, and the critical efficacy endpoints, and

to recommend whether to continue, modify, or stop a trial. The safety Committee will have password-controlled access to Follow-up webpages of the study website.

Ethics

COVID-Simile study Presentation Certificate for Ethical Appreciation (CAAE) number is 30638220.0.0000.0008, which was approved by the Brazilian National Research Ethics Commission (CONEP) on May 31st, 2020 (report # 4.059.759).

Study registration

COVID-Simile Study was registered at the University Hospital Medical Information Network (UMIN - https://www.umin.ac.jp/ctr/index.htm) on June 1st, 2020, and the trial start date was June 15, 2020. Unique ID: UMIN000040602. First participant was included on June 29,2020.

DISCUSSION

2020 ends with optimistic and pessimistic perspectives regarding the COVID-19 pandemic. The World Health Organization registers 56 COVID-19 candidate vaccines (11 in phase 3 of development)[39], but on the other hand, totalizes 75 million cases, 1.7 million deaths, and warns about increasing rates of new cases and deaths in the Americas, Europe, and Africa[40]. Moreover, the emergence of variants of SARS-CoV-2 with increased infectivity[41] raises sanitary barriers[42] and concerns about even higher morbimortality rates, until an effective global immunization is not achieved. In this scenario, safe and accessible interventions that could reduce the severity and duration of COVID-19 symptoms are urgently needed.

The search for a specific treatment for COVID-19 has driven mainstream clinical studies on antivirals, antibodies, antimalarials, etc.[43]. In homeopathic research, randomized, placebo-controlled trials are being carried out at university hospitals in Tehran[44] and New Delhi[45], respectively studying Ipecacuanha and individualized homeopathic medicines in the management of COVID-19 cases. As far as we know, this is the only double-blinded, randomized, placebo-controlled trial being conducted in Primary Care to evaluate a homeopathic strategy aiming at reducing the severity and duration of COVID-19 symptoms.

Hypothesizing *Natrum muriaticum* as able of stimulating COVID-19 "like symptoms" and COVID-19 recovery is hypothesizing effects far beyond the known biological properties of sodium chloride (NaCl), which is a characteristic of the homeopathic pharmacology: inert substances can develop pharmacological effects through dynamizing or potentizing[11]. For instance, salinization has been identified as the main process of soil degradation, but dynamized solutions of sodium chloride (i.e., *Natrum muriaticum*) can increase the production of healthy seedlings submitted to saline stress[46]. In the emerging field of nanoscience, fifty-millesimal potencies (from LM1-LM30) show "remarkable" presence of nanoparticles of Na and Cl, with contaminants observed in the LM1 degree only[47]. Those nanoparticles may be the source of a low dose of "self-similar information" that will "mobilize neural and cellular defense of the body networks"[48].

If COVID-Simile results refute the null hypothesis, exactly prepared homeopathic *Natrum muriaticum* LM2 could be incorporated into the clinical management of COVID-19 in Primary Care, in parallel with larger studies to confirm its effectiveness.

AUTHORS CONTRIBUTIONS

All authors certify that they have participated sufficiently in the work to take public responsibility for the content, including participation in the concept (UCA), design (UCA, MSA, LMH, AEMP, ATC, JNMD, CAAM, KRS, LS, HFS, EZM), analysis (EMZ), writing (UCA), or revision of the manuscript (UCA, MSA).

ACKNOWLEDGMENTS

Health Secretary of São Carlos, Mr. Marcos Palermo, for the trust. Primary Care Unities teams of São Carlos, for their collaboration in times of multiple crises. HN-Cristiano Pharmacy, São Paulo, for kindly providing the study medication. Ms. Cláudia Rejane Zangotti da Costa, Administrative Assistant at School Health Unit/UFSCar (USE), for coordinating medication and Informed Consent logistics. Thales Schiabel Adler, for reviewing the manuscript and adding relevant suggestions to the text.

DISCLOSURE STATEMENT

Cesar AT is co-owner of HN-Cristiano Homeopatia, the pharmacy that has donated the study medication, however, *Natrum muriaticum* has been in use for over 150 years and is not patentable.

FUNDING SOURCES

This research did not receive any specific grant from funding agencies in the public, commercial, or not-for-profit sectors. Study medication has been kindly provided by HN-Cristiano Homeopatia – Sao Paulo, SP, Brazil. HN-Cristiano does not participate in the study, except for the donation of study medication.

APPENDIX 1 – “COVID-SIMILE SCALE”

	COVID-Simile Scale			
	Please give a score (0, 1, 2, or 3) for each of the symptoms today			
**Symptoms**	**0**<br>*absent*	**1**<br>*light or few times a day*	**2**<br>*moderate, or a few times a day*	**3**<br>*intense, often in the day or continuously*
Shortness of breath				
Cough				
Tiredness				
Expectoration				
Headache				
Sore throat				
Chest pain				
Back pain				
Muscle aches				
Joint pain				
Lack of appetite				
Diarrhea				
Nausea				
Vomiting				
Decreased sense of smell				
Decreased sense of taste				
	0	0	0	0
Axillary temperature (highest measure on the day):			Score:	**0,0**
Date of the first symptom:				
Date of completion:				
Date of birth:				
Other symptoms today? Which?				
Adverse Event: was there any other health problem today?			No	
If yes, which?				
How many doses of *Natrum muriaticum* have you taken today?				
Other medications in use:			None	

REFERENCES

[1] WHO. Coronavirus Disease (COVID-19) Dashboard. Brazil. https://covid19.who.int/region/amro/country/br (accessed 19 December 2020).

[2] Brazil. Ministry of Health. https://covid.saude.gov.br/ (accessed 28 November 2020).

[3] Brazil. IBGE. https://cidades.ibge.gov.br/brasil/sp/sao-carlos/panorama (accessed 02 June 2020) - [Portuguese].

[4] G1 São Carlos e Araraquara. São Carlos confirms 1st case of coronavirus in 35 man; city clears 25 suspects https://g1.globo.com/sp/sao-carlos-regiao/noticia/2020/03/18/sao-carlos-confirma-1o-caso-de-coronavirus-em-homem-de-35-anos.ghtml (accessed 18 March 2020) - [Portuguese]

[5] São Carlos. City Hall/Emergency Coronavirus Combat Committee. http://coronavirus.saocarlos.sp.gov.br/ (accessed 19 December 2020) - [Portuguese]

[6] Brazil. Ministry of Health. National Policy of Integrative and Complementary Practices in SUS 2.ed. 2015. http://bvsms.saude.gov.br/bvs/publicacoes/politica_nacional_praticas_integrativas_complementares_2ed.pdf (accessed 2 June 2020) - [Portuguese]

[7] Brazil. Federal Council of Medicine. RESOLUÇÃO CFM nº 1634/2002. http://www.portalmedico.org.br/resolucoes/cfm/2002/1634_2002.htm (accessed 2 June 2020) - [Portuguese]

[8] Hahnemann S, 1921. Organon der Heilkunst: aude sapere, 6. ed. Posthumous publication by Richard Haehl. Heidelberg, Germany: Haug, 1988: § 246 [German].

[9] Hahnemann S, 1921. Organon der Heilkunst: aude sapere, 6. ed. Posthumous publication by Richard Haehl. Heidelberg, Germany: Haug, 1988: § 247-248 [German].

[10] Hahnemann S, 1921. Organon der Heilkunst: aude sapere, 6. ed. Posthumous publication by Richard Haehl. Heidelberg, Germany: Haug, 1988: § 270 [German].

[11] Hahnemann S, 1921. Organon der Heilkunst: aude sapere, 6. ed. Posthumous publication by Richard Haehl. Heidelberg, Germany: Haug, 1988: § 269 [German].

[12] Bell IR, Schwartz GE. Adaptive network nanomedicine: an integrated model for homeopathic medicine. Front Biosci (Schol Ed) 2013;5:685-708.

[13] Hahnemann S, 1921. Organon der Heilkunst: aude sapere, 6. ed. Posthumous publication by Richard Haehl. Heidelberg, Germany: Haug, 1988: § 102 [German].

[14] Hahnemann S, 1921. Organon der Heilkunst: aude sapere, 6. ed. Posthumous publication by Richard Haehl. Heidelberg, Germany: Haug, 1988: § 153 [German].

[15] Schmitt PP. Virologe Hendrik Streeck: „Wir haben neue Symptome entdeckt“. Frankfurter Allgemeine, 03/16/2020. https://www.faz.net/aktuell/gesellschaft/gesundheit/coronavirus/neue-corona-symptome-wntdeckt-virologe-hendrik-streeck-zum-virus-16681450.html (accessed 23 March 2020) - [German]

[16] Yan CH, Faraji F, Prajapati DP Boone CE, DeConde AS. Association of chemosensory dysfunction and COVID-19 in patients presenting with influenza-like symptoms. Int Forum Allergy Rhinol 2020; [Epub ahead of print] PMID: 32279441 DOI: 10.1002/alr.22579

[17] Lechien JR, Chiesa-Estomba CM, De Siati DR, et al. Olfactory and gustatory dysfunctions as a clinical presentation of mild-to-moderate forms of the coronavirus disease (COVID-19): a multicenter European study. Eur Arch Otorhinolaryngol. 2020;1-11. doi:10.1007/s00405-020-05965-1

[18] Hahnemann S. On the prevailing fever. (Original: Allgemeiner Anzeiger der Deutschen. 1809;261). Lesser Writings of Samuel Hahnemann. Collected and translated by Dudgeon RE. New Delhi: B Jain, 1984:561,564.

[19] Hahnemann S, 1921. Organon der Heilkunst: aude sapere, 6. ed. Posthumous publication by Richard Haehl. Heidelberg, Germany: Haug, 1988: § 148 [German].

[20] Möllinger H, Schneider R, Walach H. Homeopathic pathogenetic trials produce specific symptoms different from placebo. Forsch Komplementmed. 2009;16(2):105-110. doi:10.1159/000209386

[21] Miccant, Vision software https://www.miccant.com/vision.html

[22] Hahnemann S, 1921. Organon der Heilkunst: aude sapere, 6. ed. Posthumous publication by Richard Haehl. Heidelberg, Germany: Haug, 1988: § 155 [German].

[23] Hahnemann CFS. The Chronic Diseases, their Peculiar Nature and their Homœopathic Cure. Transl. Tafel LH. New Delhi: B Jain, 1986: Vol. 2 – Natrum muriaticum.

[24] Allen T. The Encyclopedia of Pure Materia Medica. New Delhi: B Jain,1988: Vol. VI – Natrum muriaticum.

[25] Brazil. Ministry of Health. Portaria 467, 03/20/2020. http://www.planalto.gov.br/CCIVIL_03/Portaria/PRT/Portaria%20n%C2%BA%20467-20-ms.htm (accessed 1 April 2020) - [Portuguese]

[26] Adler UC, Paiva NM, Cesar AT, Adler MS, Molina A, Padula AE, Calil HM. Homeopathic Individualized Q- Potencies versus Fluoxetine for Moderate to Severe Depression: Double-Blind, Randomized Non-Inferiority Trial. Evid Based Complement Alternat Med 2011; 2011:520182.

[27] Adler UC, Krüger S, Teut M, Lüdtke R, Schützler L, Martins F, Willich SN, Linde K, Witt CM. Homeopathy for depression: a randomized, partially double-blind, placebo-controlled, four-armed study (DEP-HOM). PLoS One 2013;8(9):e74537.

[28] Adler UC, Acorinte AC, Calzavara FO, da Silva AA, de Toledo Cesar A, Adler MS, Martinez EZ5, Galduróz JCF. Double-blind evaluation of homeopathy on cocaine craving: a randomized controlled pilot study. J Integr Med 2018;pii: S2095-4964(18)30039-6.

[29] Mitchiguian Hotta L, Cardinalli Adler U, de Toledo Cesar A, Martinez EZ, Demarzo MMP. Homeopathy for Perennial Asthma in Adolescents: Pilot Feasibility Study Testing a Randomised Withdrawal Design. Homeopathy 2018;107(2):143-149.

[30] Brazil. Ministry of Health. Resolução nº 196 de 10/10/1996. https://bvsms.saude.gov.br/bvs/saudelegis/cns/1996/res0196_10_10_1996.html (accessed 18 April 2020) - [Portuguese]

[31] International Council for Harmonisation of Technical Requirements for Pharmaceuticals for Human Use (ICH) ICH-GCP International Conference on Harmonisation - Good Clinical Practice - E6(R2). https://ichgcp.net/

[32] Brazil. Ministry of Health. National Health Council. Guidelines from the National Research Ethics Commission (CONEP) for conducting research during COVID (May 9th, 2020) https://drive.google.com/file/d/1apmEkc-0fe8AYwt37oQAlX90plvOja3Z/view (accessed 15 May 2020) - [Portuguese]

[33] Dean ME, Coulter MK, Fisher P, Jobst K, Walach H; Delphi Panel of the CONSORT Group. Reporting data on homeopathic treatments (RedHot): a supplement to CONSORT*. Forsch Komplementmed 2006;13(6):368-371.

[34] Adler UC, Cesar ATC, Adler MS, Padula AE. Garozzo EN. Galhardi WP. From pharmaceutical standardizing to clinical research: 20 years of experience with fifty-millesimal potencies. Int J High Dilution Res 2009; 8(29): 173-182. http://highdilution.org/index.php/ijhdr/article/view/367

[35] Zhu J, Ji P, Pang J, Zhong Z, Li H, He C, Zhang J, Zhao C. Clinical characteristics of 3,062 COVID-19 patients: a meta-analysis. J Med Virol. 2020 [Epub ahead of print]. PMID: 32293716 DOI: 10.1002/jmv.25884

[36] Butler CC, van der Velden AW, Bongard E, Saville BR, Holmes J et al. Oseltamivir plus usual care versus usual care for influenza-like illness in Primary Care: an open-label, pragmatic, randomised controlled trial. Lancet 2020;395(10217):42-52. doi: 10.1016/S0140-6736(19)32982-4. Epub 2019 Dec 12. PMID: 31839279 DOI: 10.1016/S0140-6736(19)32982-4

[37] Brazil. Ministry of Health. Secretariat of Primary Health Care. Clinical Management of Coronavirus (COVID-19) in Primary Health Care. March, 2020. https://www.saude.gov.br/images/pdf/2020/marco/20/20200318-ProtocoloManejo-ver002.pdf (accessed 1 April 2020) - [Portuguese]

[38] Kleinbaum DG & Klein M. Survival analysis. New York: Springer, 2010.

[39] WHO.R&D Blueprint. Draft landscape of COVID-19 candidate vaccines. https://www.who.int/publications/m/item/draft-landscape-of-covid-19-candidate-vaccines (accessed 20 December 2020).

[40] WHO. Coronavirus Disease (COVID-19) Dashboard. https://covid19.who.int/ (accessed 20 December 2020).

[41] Baric RS. Emergence of a Highly Fit SARS-CoV-2 Variant. N Engl J Med. 2020 Dec 16. doi: 10.1056/NEJMcibr2032888. Epub ahead of print. PMID: 33326716.

[42] Stewart H, Badshah N. Boris Johnson calls crisis meeting over new Covid strain. The Guardian, 18 December 2020. https://www.theguardian.com/world/2020/dec/18/boris-johnson-calls-crisis-meeting-to-discuss-response-to-new-covid-strain (accessed 20 December 2020).

[43] Wiersinga WJ, Rhodes A, Cheng AC, Peacock SJ, Prescott HC. Pathophysiology, Transmission, Diagnosis, and Treatment of Coronavirus Disease 2019 (COVID-19): A Review. JAMA. 2020 Aug 25;324(8):782-793. doi: 10.1001/jama.2020.12839. PMID: 32648899.

[44] Mazaherinezhad A. Double blind clinical trial study on Evaluation of the effectiveness of Ipecac homeopathic remedy in control of Clinical manifestations of COVID-19. https://en.irct.ir/trial/46736 (accessed 20 December 2020).

[45] Gupta PK. Effect of adjuvant homoeopathy with standard treatment protocol in management of covid-19: a randomised, open label, placebo controlled, parallel group study. http://www.ctri.nic.in/Clinicaltrials/pmaindet2.php?trialid=43393 (accessed 20 December 2020).

[46] Bonfim FPG, Casali VWD, Yoshikawa AM. Production of tomato seedlings using seeds pelleted with Natrum muriaticum and submitted to saline stress. International Journal of High Dilution Research 2019; 18(1):2-11.

[47] Rajendran ES, Saudi J. Nano Pharmacological Aspect of Homeopathic Drugs - A Comparative Study of Different Scales of Ultra-High Dilutions Based on HRTEM Analysis and NP Characterization of Homeopathic Drug Natrum Muriaticum 6C – CM and LM1 -LM30 Med Pharm Sci 2017;3(2):89-106.

[48] Bell IR. The Complexity of the Homeopathic Healing Response Part 1: The Role of the Body as a Complex Adaptive System in Simillimum-Initiated Recovery from Disease. Homeopathy 2020;109(2):42-50.

CORRESPONDING ADDRESS

Ubiratan Cardinalli Adler, M.D, Ph.D.

Universidade Federal de São Carlos, Medicine Department

Rodovia Washington Luiz, Km 235, São Carlos, SP, Brasil – 13565-905

Tel. +55 16 3351-9420

ubiratanadler@ufscar.br
